# Targeted alpha therapy with the ^224^Ra/^212^Pb-TCMC-TP-3 dual alpha solution in a multicellular tumor spheroid model of osteosarcoma

**DOI:** 10.3389/fmed.2022.1058863

**Published:** 2022-11-23

**Authors:** Anna Julie Kjøl Tornes, Vilde Yuli Stenberg, Roy Hartvig Larsen, Øyvind Sverre Bruland, Mona-Elisabeth Revheim, Asta Juzeniene

**Affiliations:** ^1^Department of Radiation Biology, Institute for Cancer Research, Oslo University Hospital, Oslo, Norway; ^2^ArtBio AS, Oslo, Norway; ^3^Institute of Clinical Medicine, University of Oslo, Oslo, Norway; ^4^Department of Oncology, Oslo University Hospital, Oslo, Norway; ^5^Division of Radiology and Nuclear Medicine, Oslo University Hospital, Oslo, Norway; ^6^Department of Physics, University of Oslo, Oslo, Norway

**Keywords:** targeted alpha therapy, osteosarcoma, dual alpha therapy, radium-224, lead-212, TP-3 antibody

## Abstract

Osteosarcoma patients with overt metastases at primary diagnosis have a 5-year survival rate of less than 20%. TP-3 is a murine IgG2b monoclonal antibody with high affinity for an epitope residing on the p80 osteosarcoma cell surface membrane antigen. The tumor-associated antigen p80 is overexpressed in osteosarcomas, and has very low normal tissue expression. We propose a novel dual alpha targeting solution containing two radionuclides from the same decay chain, including the bone-seeking ^224^Ra, and cancer cell-surface seeking ^212^Pb-TCMC-TP-3 for the treatment of osteoblastic bone cancers, circulating cancer cells and micrometastases. In this *in vitro* study, the cytotoxic effects of ^212^Pb-TCMC-TP-3 (single alpha solution) and ^224^Ra/^212^Pb-TCMC-TP-3 (dual alpha solution) were investigated in a multicellular spheroid model mimicking micrometastatic disease in osteosarcoma. OHS spheroids with diameters of 253 ± 98 μm treated with 4.5, 2.7, and 3.3 kBq/ml of ^212^Pb-TCMC-TP-3 for 1, 4, and 24 h, respectively, were disintegrated within 3 weeks. The ^212^Pb-TCMC-TP-3 induced a 7-fold delay in spheroid doubling time compared to a 28-times higher dose with the non-specific ^212^Pb-TCMC-rituximab. The ^224^Ra/^212^Pb-TCMC-TP-3 completely disintegrated spheroids with diameters of 218–476 μm within 3 and 2 weeks after 4 and 24 h incubation with 5 kBq/ml, respectively. Treatment with 1 kBq/ml of ^224^Ra/^212^Pb-TCMC-TP-3 for 24 h caused an 11.4-fold reduction in spheroid viability compared with unconjugated ^224^Ra/^212^Pb. The single and dual alpha solutions with TP-3 showed cytotoxicity in spheroids of clinically relevant size, which warrant further testing of the dual alpha solution using *in vivo* osteosarcoma models.

## Introduction

Osteosarcoma (OS) is the second most common bone cancer after chondrosarcoma, and the most common bone malignancy in adolescents and young adults (1, 2). The 5-year survival rate is <20% for patients with metastatic OS at primary diagnosis, and the median survival after multiple recurrences is only 1 year (3–6). Few new treatment options have been developed for metastatic OS during the past three decades, underscoring the need for novel therapies.

Immunotherapy using monoclonal antibodies (mAbs) specific for overexpressed cancer-related antigens is a promising treatment strategy for micrometastases and circulating tumor cells (7–10). Clinical trials have evaluated the efficacy of mAbs in OS, including trastuzumab targeting the human epidermal growth factor receptor 2 (HER2) (11), glembatumumab targeting glycoprotein nonmetastatic B (12), cixutumumab targeting insulin-like growth factor 1 (13, 14), and pembrolizumab, nivolumab and camrelizumab targeting PD-1 (15–19). Unfortunately, sufficient antitumor response was not demonstrated for OS patients receiving these immune-based therapies, possibly due to low expression of the tumor antigens and internal resistance mechanisms (20, 21). The murine IgG2b TP-3 mAb binds to an 80 kDa sarcoma-associated cell surface membrane antigen (p80) on an alkaline phosphatase isoform (22, 23). TP-3 is directly related to osteoblastic differentiation and has previously shown to bind to the vast majority of OS metastases in patients (23, 24). Moreover, an immunomagnetic isolation procedure using the TP-3 mAb detected single OS cells in bone marrow aspirates from OS patients that was shown to have prognostic information (24, 25). Because of the nonreactive activity of TP-3 on healthy human tissues, it is well suited for targeted therapy (22, 23).

Osteosarcoma is clinically regarded as a radioresistant cancer, and external beam radiotherapy is usually not effective for OS (26–29). Unfortunately, the mechanisms of action related to the radioresistance are not well investigated and remain unresolved (30). Targeted therapies using mAbs labeled with the beta emitting ^188^Re or ^177^Lu, and the alpha emitting ^211^At or ^213^Bi have been studied for OS *in vitro* and *in vivo* (31–38). However, beta particles have low linear energy transfer (LET, ∼ 0.2 keV/μm), making them less effective for treating radioresistant tumor cells (39). In contrast, alpha particles have a high LET (∼ 100 keV/μm) and short range in tissue (50–100 μm) compared to beta particles (0.5–12 mm), resulting in high cytotoxic potency via DNA double stranded breaks (39). Therefore, alpha particles should be preferred over beta particles to overcome radiation resistance in OS.

The alpha emitting ^223^Ra (t_1/2_ ≈ 11.4 d) is a calcium-mimetic radiopharmaceutical with naturally bone-seeking properties (40, 41). Its accumulation in osteoblastic lesions resulted in improved overall survival and approval for the treatment of castration-resistant prostate cancer patients with symptomatic bone metastases (42). Thus, ^223^Ra can also be efficient for OS patients due to the osteoblastic phenotype of this cancer (43–46). In 2021, a phase I escalation trial with 50, 75, and 100 kBq/kg of ^223^Ra was completed in 18 OS patients with progressive, locally recurrent or metastatic disease (3). The radiopharmaceutical was well tolerated and a recommended phase II dose was set to 100 kBq/kg — a twice as high dose as approved for prostate cancer, due to the high radiation tolerance (3). Unfortunately, the majority of patients developed extra-skeletal metastases (3). Since ^223^Ra cannot be stably chelated to a targeting moiety, it must be combined with other agents that can target extra-skeletal disease in these patient groups (45).

Similar to ^223^Ra, ^224^Ra (t_1/2_ ≈ 3.6 d) has the same bone-seeking properties, four alpha emissions in the decay chain and a relevant half-life for radiopharmaceutical production and shipment (47). An important difference is that ^224^Ra decays into ^212^Pb (t_1/2_ ≈ 10.6 h), which, compared to ^211^Pb (t_1/2_ ≈ 36.1 min) in the ^223^Ra-series, has a convenient half-life for conjugation to targeting molecules (47–49). Lead-212 is itself a beta emitter, but serves as an *in vivo* generator of alpha particles via its decay to ^212^Bi (t_1/2_ ≈ 60.6 min) and ^212^Po (t_1/2_ ≈ 0.3 μs). With the bifunctional 1,4,7,10-tetraaza-1,4,7,10-tetra(2-carbamoylmethyl)cyclododecane (TCMC) chelator for ^212^Pb, several radioconjugates have been produced and tested in preclinical and clinical studies (49–61). The dual alpha technology utilizes the osteoblastic stroma-seeking properties of ^224^Ra that can treat primary bone cancer or bone metastases, while extra-skeletal and skeletal metastases can be targeted by a cancer specific moiety labeled with ^212^Pb (47). In a preclinical prostate cancer study employing this technology, the biodistribution data showed high uptake of ^224^Ra in the femur and skull while a ^212^Pb-conjugate had high prostate tumor uptake (62). The technology also showed promising potential in a breast cancer study, where the ^224^Ra solution with bone targeting ^212^Pb-EDTMP [ethylenediamine tetra (methylenephosphonic acid)] prolonged survival and lowered the incidence of bone metastases in mice (63).

Representative *in vitro* models are essential to mimic tumor micrometastases. Three-dimensional (3D) multicellular spheroids have been utilized in preclinical cancer therapy studies since they exhibit physiologically relevant cell–cell and cell–matrix interactions, heterogeneity and structural complexity which better reflect cancer metastases that are found in patients (64–68). The cell–cell interactions allow contribution by the bystander and cross-fire effects to the cytotoxicity of targeted radionuclide therapy to be considered. Spheroids have a necrotic core surrounded by a viable rim of proliferating cells (69), where steep gradients can exist for cellular oxygen levels, proliferation, pH and glucose concentration (70, 71), leading to development of cell subpopulations that may be resistant to treatment, similar to tumor cells *in vivo* (72). Therefore, spheroids are advantageous *in vitro* models because overcoming these factors could not be considered in 2D monolayer models (66, 70).

In this work, the cytotoxic effect of a single alpha solution comprising ^212^Pb conjugated to the OS cell targeting mAb TP-3 and a dual alpha solution containing ^224^Ra in equilibrium with ^212^Pb-TCMC-TP-3 were evaluated using an *in vitro* 3D multicellular spheroid model.

## Materials and methods

### Cell line

The human OS cell line OHS (established at the Norwegian Radium Hospital) was used in this study (73). The OHS cell line used in the present study was obtained from a repository at the Norwegian Radium Hospital, Oslo University Hospital. The cell line was cultured in RPMI 1640 medium (Sigma-Aldrich Norway AS, Oslo) supplemented with 10% heat inactivated fetal bovine serum (FBS, GE Healthcare Life Sciences, Chicago, IL), 100 units/ml penicillin and 100 μg/ml streptomycin (Sigma-Aldrich) at 37°C with 5% CO_2_.

### Antibodies

The anti-p80 IgG2b murine mAb TP-3 was produced and purified as described by Bruland et al. (22). TP-3 (PAK-732 batch), was used in all experiments (22, 25). The chimeric anti-CD20 IgG1 mAb rituximab (RTX, MabThera, Roche, Basel) and the humanized anti-HER2 IgG1 mAb trastuzumab (TRA, Herceptin, Roche) were used as antigen negative controls.

### Flow cytometry

The expression of p80 on OHS cells was verified by flow cytometry using TP-3. The primary mAbs (TP-3 or RTX) were added to 5 × 10^5^ OHS cells in 100 μl flow buffer [Dulbecco’s PBS with 0.5% bovine serum albumin (BSA) and 0.1% NaN_3_] at a concentration of 10 μg/ml and incubated at 4°C with gentle shaking for 60 min, followed by three washes with 2 ml flow buffer. FITC-conjugated anti-mouse IgG F(ab’)2 fragment (Thermo Fisher Scientific, Waltham, MA) was used as a secondary Ab and added at a concentration of 10 μg/ml, incubated at 4°C with gentle shaking for 30 min in the dark and washed as in the previous step. All wash steps were performed by centrifugation at 260 × *g* for 5 min. Washed cell pellets were resuspended in 200 μl flow buffer and analyzed by a Cytoflex S flow cytometer (Beckman Coulter, Inc., Brea, CA) using the CytExpert 2.0 software (Beckman Coulter, Inc.) for data acquisition. The FlowJo software (FlowJo, LLC, Ashland, OR) was used for data analysis.

### Preparation of radionuclides and activity measurements

Radium-224 was extracted from a generator column containing DIPEX^®^ actinidine resin (Eichrom Technologies, Lisle, IL) with immobilized ^228^Th (Eckert & Ziegler, Braunschweig) by elution with 1 M HCl. The details of the ^224^Ra generator setup have previously been described by Westrøm et al. and Stenberg et al. (62, 74). Lead-212 was produced and extracted from a ^228^Th generator, via emanation of ^220^Rn in a simplified single-chamber generator system, described by Li et al. (75). Lead-212 was extracted from the flask using 0.1 M HCl, with a ^228^Th breakthrough ≤0.005%. The collector flask was replaced 2–3 days prior to the experiments to prevent accumulation of ^208^Pb. A Capintec CRC-25R radioisotope dose calibrator (Capintec Inc., Ramsey, NJ) was used to quantify the ^224^Ra and ^212^Pb activities (76).

A Hidex automatic gamma counter (Hidex Oy, Turku) or Cobra gamma counter (Packard Instrument Company, Downer Grove, IL) with the 50–120 keV counting window was used to determine ^212^Pb activities. The counting window mainly measures ^212^Pb activity (34.9% relative to the ^224^Ra mother nuclide) with only small contribution from other radionuclides (1.2% relative to ^224^Ra) in the ^224^Ra series (47, 62, 74, 77). All samples were measured at least 2 h after ^212^Pb extraction to ensure transient equilibrium with daughters. The activity of ^224^Ra was determined by measurements performed 4–5 days after the experiment, when ^212^Pb had decayed and equilibrium between ^224^Ra and the newly formed ^212^Pb was reached.

### Radiolabeling of antibodies

The mAbs were conjugated to a TCMC chelator (Macrocyclics Inc., Dallas, TX), to allow radiolabeling with ^212^Pb. The original buffer of the mAbs was first exchanged with carbonate buffer (0.1 M NaHCO_3_ and 5 mM Na_2_CO_3_ in metal free water) by washing the mAb solution through a centrifugal concentrator (30 kDa, Amicon Ultra-15 Centrifugal Filter Unit, Millipore, Sigma-Aldrich) three times at 1620 × *g* for 15–25 min. A TCMC solution in 5 mM HCl was added to the Abs in a 5:1 molar ratio, and the mixture were allowed to react for 2 h with gentle shaking (250 min^–1^) at room temperature. Then, unconjugated TCMC was removed from TCMC-mAb conjugates by exchanging the carbonate buffer to 0.9% NaCl by washing the mAb solution through the centrifugal concentrator as described above. The concentration of the mAbs was quantified by Nanodrop (Nanodrop 1000 Spectrophotometer, V3.8, Thermo Fisher Scientific), using the standard absorbance value of IgG at 280 nm and 10 mm path length.

The mAbs were labeled with ^212^Pb using ^224^Ra or ^212^Pb solutions (pH adjusted to 5–6 by 0.5 M C_2_H_7_NO_2_ or C_2_H_3_NaO_2_) in equilibrium with daughters. TCMC-TP-3, TCMC-RTX, or TCMC-TRA was added to a final concentration of 0.1–1 mg/ml. The solutions were mixed on a Thermomixer (Eppendorf, Hamburg) for 45 min at 37°C and 450–750 rpm. Radiochemical purity of the samples was determined by instant thin layer chromatography (Tec-control, Biodex, Medical Systems, Shirley, NY), and only products with purities ≥95% were used in the experiments. The final solution consisting of ^212^Pb-TCMC-mAb is referred to as the “single alpha solution” while ^212^Pb-labeled mAbs in the presence of ^224^Ra is called the “dual alpha solution.”

### Saturation binding studies

OHS cells were detached from a cell culture flask using TrypLE Express (Sigma-Aldrich). Saturation binding studies of ^212^Pb-TCMC-TP-3 to OHS cells were performed by collecting 10^6^ of the cells and incubating them as cell suspension in 0.2 ml of PBS including 0.5% BSA (Sigma-Aldrich) with 6 different concentrations (0.03–10 μg/ml) of the radioimmunoconjugate (in duplicates) for 1 h at 37°C and 150 min^–1^. Non-specific binding was measured by pre-incubating cells with unlabeled TP-3 (5–20 μg/ml) for 15 min before addition of ^212^Pb-TCMC-TP-3. Activities were measured in a gamma counter before (added activity) and after incubated cells were washed 3 times with PBS containing 0.5% BSA (cell bound activity). Specific cell bound activity was estimated as percentage of added activity minus non-specific binding (activity on blocked cells). The number of specifically bound ligands per cell was plotted against ligand concentration and the equilibrium dissociation constant (K_*D*_) and the number of specific binding sites (Bmax), were determined by nonlinear regression (Sigmaplot version 14.5, Systat Software, Inc., San Jose, CA, USA).

### Spheroid formation and treatment

Multicellular tumor spheroids were generated using the liquid-overlay technique (78, 79). Spheroids were formed by seeding 500 OHS cells in 100 μl of culture medium per well in a 96-well flat-bottom plate coated with 50 μl of 1.5% agarose (weight/volume, Sigma-Aldrich) solution in PBS (Sigma Aldrich). The plates were centrifuged at 470–1000 × *g* for 15 min, and maintained at 37°C with 5% CO_2_.

For the single alpha solution studies, spheroids were treated 4–5 days after formation (day 0) with 0.3–100 kBq/ml of ^212^Pb-TCMC-TP-3 (specific activities of 7.3–15.4 MBq/mg) or ^212^Pb-TCMC-RTX (specific activities of 7.5–9.9 MBq/mg). For the dual alpha solution studies, 0.3–10 kBq/ml of ^224^Ra/^212^Pb, ^224^Ra/^212^Pb-TCMC-TP-3 (specific activities of 2.3–9 MBq/mg), or ^224^Ra/^212^Pb-TCMC-RTX/TRA (specific activities of 3.7–9 MBq/mg) were added 3 or 14 days after spheroids were formed. One experiment with TP-3 alone (0.25 μg per spheroid 2.1 μg/ml) was performed in OHS spheroids to investigate the cytotoxicity of the mAb itself. The spheroids were incubated with the mAb alone or with radioconjugates at 37°C for 1, 4, or 24 h before they were carefully washed 6 times with medium and further incubated for 3 weeks. Replacement of culture medium was performed 2–3 times per week. The spheroid diameter (d) was measured weekly using an inverted Axiovert 200M microscope (Carl Zeiss AG, Jena, Germany). Volumes of each spheroid were calculated using the formula$V=\frac{4}{3}\,\pi \,r^{3}$, where *r* = d/2. The spheroid volumes were normalized by dividing the volumes at different activities by the volume at 0 kBq/ml at each time point (activity vs. normalized volume), and by dividing the volumes of the treated spheroids at each week by the volume of spheroids at day 0 for each activity concentration (time vs. normalized volume).

A CellTiter-Glo^®^3D cell viability assay (Promega Corporation, Madison, WI, USA), was performed 48 h, 72 h and 12 days after spheroids were administered with ^224^Ra/^212^Pb, ^224^Ra/^212^Pb-TCMC-TP-3 and ^224^Ra/^212^Pb-TCMC-TRA for 24 h according to the manufacture’s protocol.

Live and dead cells in the spheroids were determined by a fluorescence-based staining assay using fluorescein diacetate (FDA, 5 mg/ml, Sigma-Aldrich) and propidium iodide (PI, 2 mg/ml, Sigma-Aldrich), respectively. Fluorescent images of spheroids were taken by an inverted Axiovert 200M microscope (Carl Zeiss AG) and analyzed with the AxioVision Rel. 4.8 software (Carl Zeiss AG). The experiment was performed with 3–12 spheroids per treatment condition. The relative viability was calculated by dividing the viability of the treated spheroid by the viability of the control spheroid (0 kBq/ml) at different activity concentrations.

Complete growth inhibition of a spheroid was considered when no live cells were detected in the spheroid, or when the spheroid diameter was reduced or remained unchanged, or when the spheroid was disintegrated/fell apart.

### Statistical analyses

SigmaPlot 14.5 (Systat Software) was used for the statistical analyses. Nonlinear regression with one-site saturation ligand binding was used to estimate the number of specific binding sites (B_max_) and the equilibrium dissociation constant (K_D_) for ^212^Pb-TCMC-TP-3 on the OHS cells:


$$B=\frac{B\,m\,a\,x\times \left[A\,b\right]}{K_{D}+\left[A\,b\right]}$$

where B is the number of antigens per cell and [Ab] is the Ab concentration. Exponential decay fitting was used to plot the correlation between the radioactivity and spheroid volume.

The spheroid volumes and viabilities were analyzed for significance using a one-way ANOVA with multiple comparisons and a pairwise *t*-test, respectively, using SigmaPlot 14.5 (Systat Software). A *p*-value of < 0.05 was considered statistically significant.

## Results

### Binding of TP-3

**FIGURE 1:**
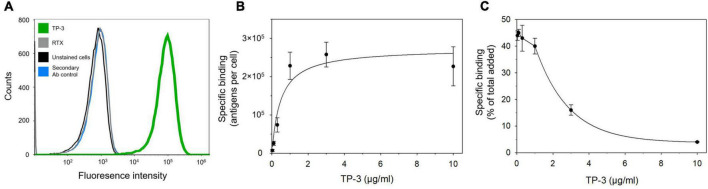
p80 expression and specific binding of TP-3 to OHS human osteosarcoma cells. **(A)** A flow cytometry histogram that demonstrates the binding of TP-3 to the p80 antigen (green curve), compared to the non-specific primary antibody rituximab (RTX; gray curve), OHS cells stained only with the secondary antibody (blue curve) or unstained control cells (black curve). **(B)** Specific binding (antigens per cell) and **(C)** percentage specific bound TP-3 (of total added) to OHS cells after 1 h incubation with 0.03–10 μg/ml of ^212^Pb-TCMC-TP-3.

A high expression of p80 was confirmed by a well-defined histogram shape of TP-3, which demonstrated binding to 99.7% of the OHS cells with no overlap and clear separation from the histograms representing the control samples (Figure 1A). Negligible non-specific binding was detected (< 0.06%) as the histogram of RTX was identical as the unstained cells and the secondary Ab control.

The OHS cells showed an average of 2.7 ± 0.3 × 10^5^ binding sites per cell (B_max_). The ^212^Pb-TCMC-TP-3 demonstrated increased binding to OHS cells at 0.03–1 μg/ml after 1 h with a K_D_ of 0.49 ± 0.05 μg/ml (Figure 1B). Similar levels of ^212^Pb-TCMC-TP-3 were bound to the OHS cells at 4 h (Supplementary Figure 1). From 1 to 10 μg/ml, the percentage of specific bound ^212^Pb-TCMC-TP-3 was reduced due to saturated binding sites (Figure 1C). Of total added radioimmunoconjugate, 5.7–12.2% was internalized to the cells after 1 h (Supplementary Figure 1).

### Cytotoxicity of the ^212^Pb-TCMC-TP-3 single alpha solution

**FIGURE 2:**
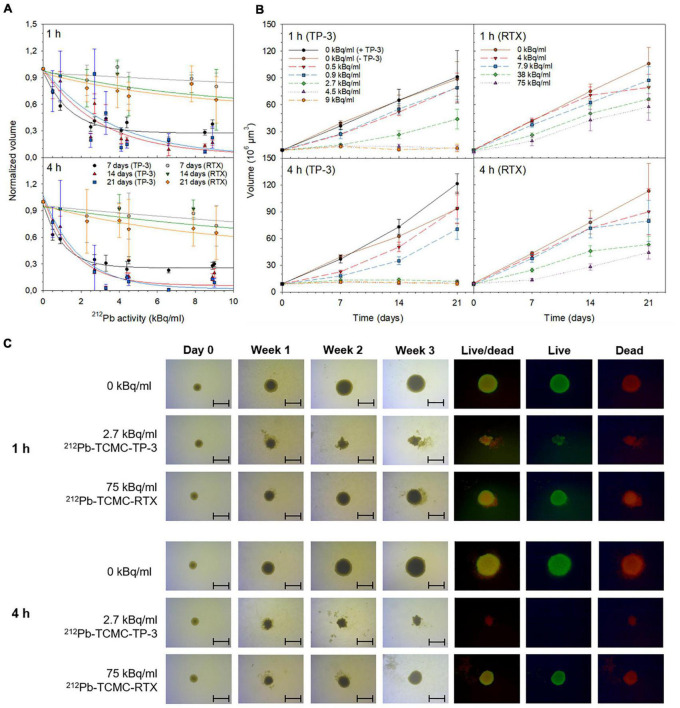
Cytotoxicity of single alpha solutions. The influence of ^212^Pb-TCMC-TP-3 or ^212^Pb-TCMC-rituximab (RTX) on OHS spheroid growth after 1 or 4 h treatment at **(A)** increasing ^212^Pb-activities and **(B,C)** over time. The normalized volume was calculated by dividing the volume of the treated spheroid by the volume of untreated spheroids (0 kBq/ml) at each time point. **(C)** Microscope images (4× magnification, scale bar = 500 μm) were taken from the day of treatment (day 0, 8.5 ± 0.5 × 10^6^ μm^3^) to week 3. At the experimental end point (week 3), spheroids were stained with fluorescein diacetate and propidium iodide to observe live and dead cells, respectively. All spheroid images were taken by an inverted Axiovert 200M microscope (Carl Zeiss AG) and analyzed with the AxioVision Rel. 4.8 software (Carl Zeiss AG). Specific activities were 7.3–15.4 MBq/mg.

The spheroids had a diameter of 253 ± 98 μm and a volume of 8.5 ± 0.5 × 10^6^ μm^3^ at day 0 (treatment day). The majority of OHS cells in these spheroids were viable at this time point (Supplementary Figure 6). The volume of spheroids decreased with increasing ^212^Pb-TCMC-TP-3 activity (Figure 2A and Supplementary Figure 2). At 3 weeks, 1 and 4 h incubation with 2.7–9 kBq/ml of ^212^Pb-TCMC-TP-3 significantly inhibited the spheroid growth and reduced the number of live cells compared to the control (*p* < 0.005, Figures 2B,C, Supplementary Figures 3, 4, and Supplementary Table 3). The doubling time was 7-fold longer for the spheroids treated with 2.7 kBq/ml of ^212^Pb-TCMC-TP-3 for 4 h compared to the control, while 6.3-fold longer than for the spheroids treated with 75 kBq/ml of ^212^Pb-TCMC-RTX (Supplementary Table 1). After 3 weeks, no live cells or spheroid growth were detected in the spheroids treated with ≥ 2.7 kBq/ml of ^212^Pb-TCMC-TP-3 for 4 and 24 h (Figure 2C and Supplementary Figures 4, 5). The non-specific ^212^Pb-TCMC-RTX was only able to completely inhibit spheroid growth after 3 weeks when treated with the high activity dose of 72.8 kBq/ml for 24 h (Supplementary Figure 5).

### Cytotoxicity of the ^224^Ra/^212^Pb-TCMC-TP-3 dual alpha solution

**FIGURE 3:**
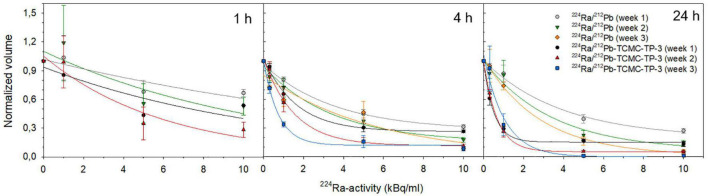
Cytotoxicity of dual alpha solutions. The influence of 1, 4, or 24 h incubation of ^224^Ra/^212^Pb and ^224^Ra/^212^Pb-TCMC-TP-3 on OHS spheroid growth at increasing ^224^Ra/^212^Pb-activities. The normalized volume was calculated by dividing the volume of the treated spheroid by the volume of untreated spheroids at each week. Spheroids were 5.8–60 × 10^6^ μm^3^ at day 0. Specific activities were 2.3–9 MBq/mg.

The cytotoxicity of the dual alpha solutions was investigated in small (diameter of 222 ± 14.0 μm and volume of 5.8 ± 1.0 × 10^6^ μm^3^ at day 0) and large (diameter of 474 ± 2 μm and volume of 60 ± 5.0 × 10^6^ μm^3^ at day 0) spheroids. The majority of OHS cells in the small spheroids were viable, while the large spheroids developed central necrosis with a 20–30 μm rim of viable cells (Supplementary Figure 6). All spheroid volumes were reduced at increasing activities, and the growth of spheroids treated for 4 or 24 h with ≥ 5 kBq/ml of ^224^Ra/^212^Pb-TCMC-TP-3 was completely inhibited (Figure 3 and Supplementary Figure 7).

Over time, the volume of spheroids treated with 1 kBq/ml of ^224^Ra/^212^Pb or ^224^Ra/^212^Pb-TCMC-RTX for 4 h increased, whereas this activity concentration of ^224^Ra/^212^Pb-TCMC-TP3 inhibited spheroid growth (Supplementary Figure 7). The doubling time of spheroids treated with 10 kBq/ml of ^224^Ra/^212^Pb-TCMC-TP-3 for 1 h was 101 days, while it was 15 and 22 days for spheroids treated with ^224^Ra/^212^Pb and ^224^Ra/^212^Pb-TCMC-RTX, respectively (Supplementary Table 2).

**FIGURE 4:**
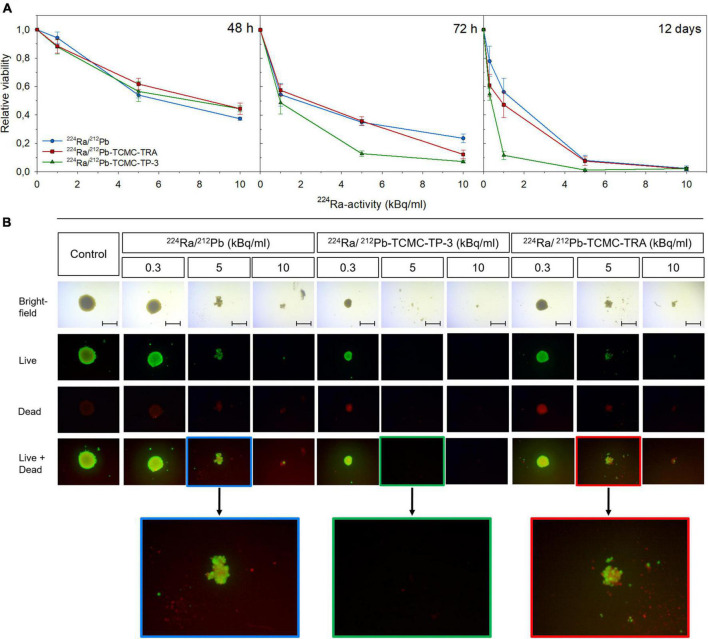
Viability of OHS spheroids treated with dual alpha solutions. **(A)** Viability of spheroids 48 h, 72 h, and 12 days after 24 h incubation with 1–10 kBq/ml of ^224^Ra/^212^Pb, ^224^Ra/^212^Pb-TCMC-TP-3 (specific activity of 2.3 MBq/mg) or ^224^Ra/^212^Pb-TCMC-trastuzumab (TRA, specific activity of 3.7 MBq/mg). The relative viability was calculated by dividing the viability of the treated spheroids by the viability of untreated spheroids (0 kBq/ml) at each activity concentration. **(B)** At day 12, spheroids were stained with fluorescein diacetate and propidium iodide to observe live and dead cells, respectively, before spheroids were imaged (4× magnification) using an inverted Axiovert 200M microscope (Carl Zeiss AG, Jena, Germany), scale bar = 500 μm. All spheroids were analyzed with the AxioVision Rel. 4.8 software (Carl Zeiss AG). Spheroids were 60 ± 5.0 × 10^6^ μm^3^ at day 0.

A viability assay was performed for spheroids treated with the dual alpha solutions for 24 h. At 72 h, a significant reduction in viability was seen in the spheroids treated with 5 kBq/ml of ^224^Ra/^212^Pb-TCMC-TP-3 versus ^224^Ra/^212^Pb or ^224^Ra/^212^Pb-TCMC-TRA (*p* < 0.05, Supplementary Table 4). Twelve days after treatment, the relative viabilities of the spheroids treated with 1 kBq/ml of the ^224^Ra/^212^Pb-TCMC-TP-3,^224^Ra/^212^Pb and ^224^Ra/^212^Pb-TCMC-TRA dual alpha solutions were 11.6 ± 2.7, 56.2 ± 6.6 and 47.2 ± 6, respectively (Figure 4A).

Complete disintegration was observed for all spheroids incubated for 24 h with 10 kBq/ml of any dual alpha solution. However, only spheroids treated with the ^224^Ra/^212^Pb-TCMC-TP-3 dual alpha solution were disintegrated 12 days after incubation with 5 kBq/ml (Figure 4B), while the relative viability of spheroids treated with ^224^Ra/^212^Pb and ^224^Ra/^212^Pb-TCMC-TRA were 8.0 ± 3.4 and 7.4 ± 2.4 at this time point, respectively.

## Discussion

The treatment options for advanced OS after standard regimen are limited due to chemotherapeutic resistance (80–82). Micrometastases are found in the majority of patients with advanced and/or recurrent OS (25, 83). Patients with micrometastases in the bone marrow or peripheral blood, also from other forms of cancer than OS, have shown statistically poorer survival compared to patients without micrometastases (25, 82, 84–88). The size of spheroids used in the present study was chosen based on a previous study by Däster et al. (89). In agreement with the study, the small spheroids herein consisted almost entirely of viable cells, while the large spheroids had a necrotic core surrounded by a rim of viable cells (Supplementary Figure 6). The single and dual alpha solutions containing TP-3 were able to disintegrate spheroids with diameters ranging from 210 to 480 μm, which is similar to the diameter of micrometastatic clusters found in patients (250–750 μm) (65, 90, 91). As already mentioned, TP-3 has previously shown the ability to detect single micrometastatic cells in bone marrow aspirates from OS patients, in which a strong correlation was detected between the TP-3 bound OS cells and poor therapeutic response following relapse (24, 25). In the present study, the TP-3 mAb itself did not initiate any cytotoxicity on OHS multicellular spheroids, which is in agreement with a previous study (33).

In contrast, the cytotoxic effect was significantly improved when spheroids were treated with ≥ 2.7 kBq/ml of the ^212^Pb-TCMC-TP-3 radioimmunoconjugate (*p* < 0.005, Figure 2 and Supplementary Table 3). The therapeutic efficacy of ^211^At-TP-3 was extensively explored three decades ago and showed promising potential for OS *in vitro* and *in vivo*, similar to a more recent study evaluating a ^213^Bi radioimmunoconjugate (33–35, 38, 92). Unfortunately, challenges related to the production of ^211^At and the very short half-life of ^213^Bi limits the applicability of these radionuclides in targeted therapy. TP-3 immunotoxins have previously showed to be effective in clonogenic OS cells *in vitro*, and in a subcutaneous as well as in a soft-tissue sarcoma model *in vivo* (93–95). SCID mice xenografted with OHS cells were treated with 1.0 mg/kg of a TP-3 immunotoxin and 67 ± 19% of the mice were tumor-free at 150 days, compared to the control mice which had a median survival of 19 days (94). Despite these encouraging data, immunogenicity and nonspecific toxicity is known to limit the clinical success of immunotoxins (96). Nevertheless, TP-3 seems highly promising for targeted therapies because of the high expression levels of p80 on the OS cell surface and the limited normal-tissue distribution (22, 23).

An important advantage with the 3D spheroid model is the possibility to observe the treatment response over an extended period, and thereby observe the repopulation potential of surviving cells. After 1 week, the volume of spheroids treated with 2.7 kBq/ml of ^212^Pb-TCMC-TP-3 for 1 h looked similar as spheroids treated with 4.5 and 9 kBq/ml (Figure 2B). Yet, regrowth of the spheroids was seen from week 2. After 4 h incubation with 2.7 kBq/ml of the ^212^Pb-TCMC-TP-3 single alpha solution, spheroids were disintegrated and no viable cells were observed at week 3 (Figure 2). This cytotoxic effect is similar to a study investigating comparable activities of ^212^Pb in 3D prostate cancer spheroids (52). A study by Ballangrud et al. investigated the therapeutic effect of a ^213^Bi labeled mAb in multicellular prostate cancer spheroids with a diameter of 200 μm, and showed that the penetration time of the radioimmunoconjugate was around 3 h (97). This supports the results observed herein, where a substantial increased cytotoxic effect was seen in spheroids (diameter of 253 ± 98 μm) treated with 2.7 kBq/ml of ^212^Pb-TCMC-TP-3 when the treatment duration increased from 1 to 4 h (Figure 2). These results are also in agreement with Hjelstuen et al. who demonstrated that 6 h was required for the ^125^I labeled mAb to reach the inner center of an OHS spheroid with a diameter of 400–450 μm (98). Nevertheless, because of the short half-life of ^213^Bi (t_1/2_ ≈ 46 min), 76% of the decay in Ballangrud’s study had already occurred by the time that the radioimmunoconjugate reached the inner core of the spheroid (97). This highlights the benefit of labeling a targeting molecule with ^212^Pb, as it has a long enough half-life for the majority of the deposited energy from the total decay to be emitted within the spheroid. However, it is important to mention that the OS tumor microenvironment is highly complex and the penetration time will also depend on other factors such as density and vascularity enabling diffusion of the targeting complex (70).

The growth inhibition of spheroids observed 3 weeks after 4 h treatment with 2.7–4.5 kBq/ml of the ^212^Pb-TCMC-TP-3 single alpha solution (Figure 2 and Supplementary Figure 4) and 5 kBq/ml of the ^224^Ra/^212^Pb-TCMC-TP-3 dual alpha solution (Figure 3 and Supplementary Table 2) corresponds to 13.5–22.5 MBq of ^212^Pb and 25 MBq of ^224^Ra per patient (∼ 5 L blood). Subbiah et al. recommended an activity of 100 kBq/kg of ^223^RaCl_2_ for the phase II study in OS patients (3). Assuming similar clinical dosing of ^224^Ra as for ^223^Ra (taken the different half-lives into account), this would translate into a clinically relevant dosage of 315 kBq/kg of ^224^Ra. In equilibrium with daughters, the ^212^Pb-immunoconjugate in the dual alpha solution would then be given in a dosage of approximately 24 MBq per patient (∼ 70 kg). This falls within the activity range of the ^212^Pb-TCMC-TRA radioimmunoconjugate (13–47 MBq) that was evaluated as safe for patients with HER2 overexpressing intraperitoneal cancer (55). This suggests that ^224^Ra/^212^Pb-TCMC-TP-3 induces cytotoxic effects at clinically relevant activity doses, although activity conversions from mouse to human also may be based on body surface area and not only on radioactivity per gram of bodyweight or blood (99).

Limitations of this study include the use of non-osteoblastic OHS spheroids, leading to an insignificant improvement in cytotoxicity from the naturally bone-seeking ^224^Ra itself. Therefore, the dual alpha solution with TP-3 required similar activities to disintegrate spheroids as the single alpha solution, meaning that the antitumor effect of the multicellular spheroids was likely linked to the delivery of the radiation emitted from the specifically bound ^212^Pb-TCMC-TP-3. Preincubation of OS cells in a calcifying medium can force the cells into an osteoblastic-like state, which will enhance the similarity of a true OS microenvironment such as observed in patients, and thereby potentially initiate an effect of ^224^Ra (100). This should be evaluated in future studies using multicellular spheroid models of OS. Furthermore, following the protocol of Jacques et al., establishing a murine model with bone sarcoma would be the next step to fully explore the potential of the dual alpha *in vivo* (101). Nonetheless, previous dual alpha studies have verified that ^224^Ra accumulates in bone (62, 63). In fact, in a preclinical breast cancer study, a ^224^RaCl_2_ solution with the ^212^Pb-daughter chelated to the bone-seeking EDTMP demonstrated similar therapeutic effect as a comparable *in vivo* study with ^223^RaCl_2_, but with significantly fewer radium atoms (47, 63, 102). A ^224^Ra solution without a chelating agent for the lead-daughter, such as for the approved ^223^RaCl_2_, will also have bone-seeking properties, but since ^212^Pb have a half-life that allows trans-organ redistribution if let free in physiological liquids like blood, saliva or lymphatic liquid, the toxicity impact from ^212^Pb is important to consider and will be minimized by the conjugation to TP-3 or EDTMP. An experimental limitation in this study includes adding activities from the single and dual alpha solutions to spheroids of different size. Ideally, the single and dual alpha experiments should be performed simultaneously with similar spheroid size at day 0. This should be considered in future studies.

## Conclusion

This study demonstrated high cytotoxicity at clinically relevant activities of the single and dual alpha solutions in spheroids mimicking micrometastatic OS disease. These results warrant further exploration in preclinical models to evaluate the therapeutic efficacy and cytotoxicity of the ^224^Ra/^212^Pb-TCMC-TP-3 dual alpha solution in an osteoblastic OS environment.

## Data availability statement

The original contributions presented in this study are included in the article/Supplementary material, further inquiries can be directed to the corresponding author.

## Author contributions

AJKT, AJ, ØSB, and RHL: conceptualization. AJKT, AJ, VYS, ØSB, and RHL: designing the work. AJKT and AJ: *in vitro* experiments. AJKT, AJ, and VYS: analyzing the data. AJ, AJKT, VYS, ØSB, M-ER, and RHL: interpretation of results and drafting the manuscript and revising it critically for important intellectual content. All authors read and agreed to the published version of the manuscript.
