# Supplementary material for: Targeted alpha therapy with the 224Ra/212Pb-TCMC-TP-3 dual alpha solution in a multicellular tumor spheroid model of osteosarcoma
Source: Front Med (Lausanne). 2022 Nov 23;9:1058863. doi: 10.3389/fmed.2022.1058863 (PMC9727293; doi:10.3389/fmed.2022.1058863)
Supplement: Supplementary file 1 [file Data_Sheet_1.pdf]

Supplementary Material

1 Supplementary Figures and Tables

Table S1. Doubling time of spheroids (8.5 ± 0.5 × 10^6 µm^3 at day 0) treated with 0.5–9 kBq/ml of ^{212}Pb-TCMC-TP-3 (specific activities of 7.3–15.4 MBq/mg) or 3.9–75 kBq/ml of ^{212}Pb-TCMC-rituximab (RTX) (specific activities of 7.5–9.9 MBq/mg). D, complete disintegration of spheroid. The doubling times corresponds to the spheroid volumes illustrated in Figure 2.

**Activity (kBq/ml)**		**0**	**0.5**	**0.9**	**2.7**	**4.5**	**9.0**
**^{212}Pb-TCMC-TP3**	**1 h**	9	8	8	9	(D)	(D)
	**4 h**	9	7	7	63	(D)	(D)
**Activity (kBq/ml)**		**0**	**3.9**	**7.8**	**37.5**	**75**	
**^{212}Pb-TCMC-RTX**	**1 h**	8	11	9	9	8	
	**4 h**	8	9	10	10	9	

Table S2. Doubling time of large spheroids (60 ± 5.0 × 10^6 µm^3 at day 0) treated with 0.3–10 kBq/ml of the dual alpha solutions (specific activity of 9 MBq/mg). N/A, no data was obtained; D, all or the majority of spheroids were disintegrated. The doubling times corresponds to the spheroid volumes illustrated in Figure 3.

**Activity (kBq/ml)**		**0**	**0.3**	**1.0**	**5.0**	**10.0**
**^{224}Ra/^{212}Pb**	**1 h**	8	N/A	8	15	15
	**4 h**	8	11	10	35	(D)
	**24 h**	8	7	10	53	(D)
**^{224}Ra/^{212}Pb-TCMC-TP-3**	**1 h**	8	N/A	8	33	101
	**4 h**	8	8	30	(D)	(D)
	**24 h**	8	10	15	(D)	(D)
**^{224}Ra/^{212}Pb-TCMC-RTX**	**1 h**	8	N/A	8	35	22
	**4 h**	8	11	10	19	(D)
	**24 h**	8	7	13	(D)	(D)

Table S3. Summary of p-values from the single alpha spheroid study at week 3 (Figure 2B), obtained by performing an ANOVA test on all experimental groups at 1 or 4 h. Groups were considered significantly different when $p < 0.05$.

		**1 h**	**4 h**
**$^{224}Ra/^{212}Pb$-TCMC-TP-3**	0 kBq/ml (-TP-3) vs. 0 kBq/ml (+TP-3)	1	1
	0 kBq/ml (-TP-3) vs. 0.5 kBq/ml	1	1
	0 kBq/ml (-TP-3) vs. 0.9 kBq/ml	1	1
	0 kBq/ml (-TP-3) vs. 2.7 kBq/ml	**0.002**	**0.003**
	0 kBq/ml (-TP-3) vs. 4.5 kBq/ml	**< 0.001**	**< 0.001**
	0 kBq/ml (-TP-3) vs. 9 kBq/ml	**< 0.001**	**< 0.001**
	0 kBq/ml (+TP-3) vs. 0.5 kBq/ml	1	1
	0 kBq/ml (+TP-3) vs. 0.9 kBq/ml	1	1
	0 kBq/ml (+TP-3) vs. 2.7 kBq/ml	0.138	**0.004**
	0 kBq/ml (+TP-3) vs. 4.5 kBq/ml	**0.001**	**0.002**
	0 kBq/ml (+TP-3) vs. 9 kBq/ml	**0.002**	**0.001**
	0.5 kBq/ml vs. 0.9 kBq/ml	1	1
	0.5 kBq/ml vs. 2.7 kBq/ml	0.471	0.616
	0.5 kBq/ml vs. 4.5 kBq/ml	**0.007**	0.331
	0.5 kBq/ml vs. 9 kBq/ml	**0.010**	0.271
	0.9 kBq/ml vs. 2.7 kBq/ml	**0.026**	**0.006**
	0.9 kBq/ml vs. 4.5 kBq/ml	**< 0.001**	**0.009**
	0.9 kBq/ml vs. 9 kBq/ml	**< 0.001**	**0.006**
**$^{224}Ra/^{212}Pb$-TCMC-RTX**	0 kBq/ml vs. 4 kBq/ml	0.089	0.326
	0 kBq/ml vs. 7.9 kBq/ml	0.267	0.145
	0 kBq/ml vs. 38 kBq/ml	**0.005**	**0.006**
	0 kBq/ml vs. 75 kBq/ml	**0.001**	**0.002**
	4 kBq/ml vs. 7.9 kBq/ml	0.424	0.716
	4 kBq/ml vs. 38 kBq/ml	0.492	0.127
	4 kBq/ml vs. 75 kBq/ml	0.214	**0.038**
	7.9 kBq/ml vs. 38 kBq/ml	0.237	0.291
	7.9 kBq/ml vs. 75 kBq/ml	0.066	0.134
	38 kBq/ml vs. 75 kBq/ml	0.649	0.565

Table S4. Summary of p-values from the viability study (Figure 4A), obtained by performing a t-test on each pair of experimental groups. Groups were considered significantly different when $p < 0.05$. N = 1.

	Activity (kBq/ml)	$^{224}Ra/^{212}Pb$ vs. $^{224}Ra/^{212}Pb$-TCMC-TP-3	$^{224}Ra/^{212}Pb$ vs. $^{224}Ra/^{212}Pb$-TCMC-TRA	$^{224}Ra/^{212}Pb$-TCMC-TP-3 vs. $^{224}Ra/^{212}Pb$-TCMC-RTX
**48 h**	**1**	0.083	**0.027**	0.828
	**5**	0.549	**0.014**	0.281
	**10**	**< 0.001**	**0.046**	0.984
**72 h**	**1**	0.343	0.586	0.141
	**5**	**0.029**	0.707	**0.029**
	**10**	**0.001**	**0.044**	**0.029**
**12 d**	**0.3**	0.100	0.100	0.100
	**1**	**0.001**	**0.029**	**0.002**
	**5**	0.083	0.101	**0.048**
	**10**	0.390	0.172	0.116

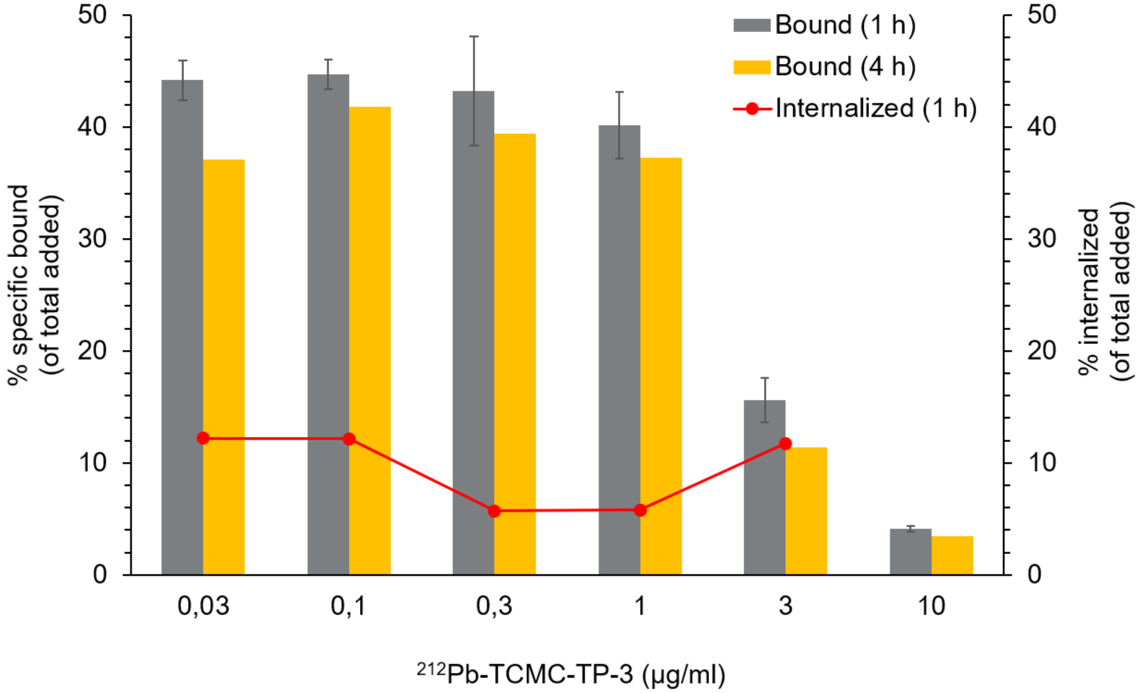

Figure S1. Percentage specific bound ^{212}Pb-TCMC-TP-3 of total added activity after 1 (N = 3) and 4 hours (N = 1) incubation, and percentage internalized ^{212}Pb-TCMC-TP-3 of total added activity after 1 h incubation (N = 1) at 0.03–10 μg/ml in OHS cells.

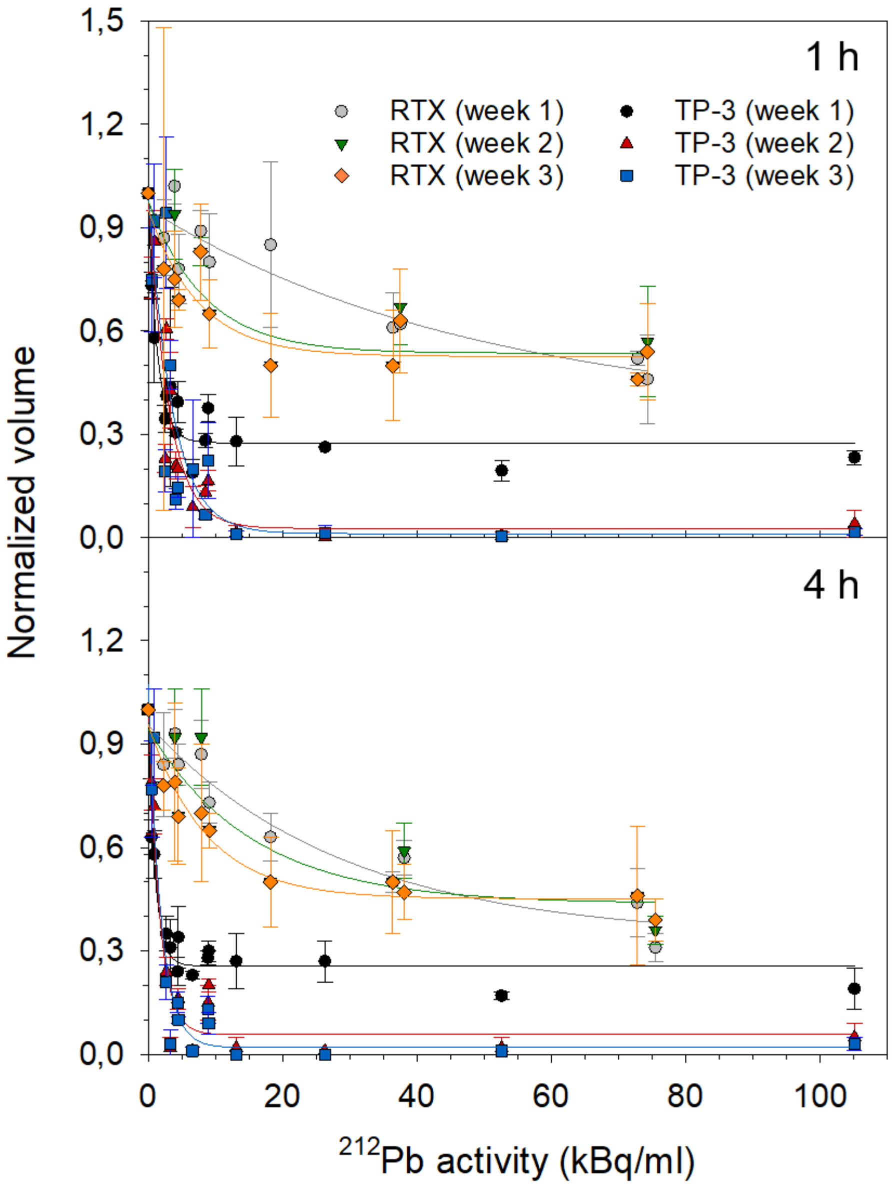

Figure S2. The influence of ^{212}Pb-TCMC-TP-3 or ^{212}Pb-TCMC-rituximab (RTX) single alpha solutions on OHS spheroid growth after treatment for 1 or 4 h with increasing ^{212}Pb-activities. The spheroids were $8.5 \pm 0.5 \times 10^6$ µm^3 at the day of treatment (day 0). The normalized volume was calculated by dividing the volume of the treated spheroid at each activity concentration by the volume of untreated spheroids at different time points (week 1–3).

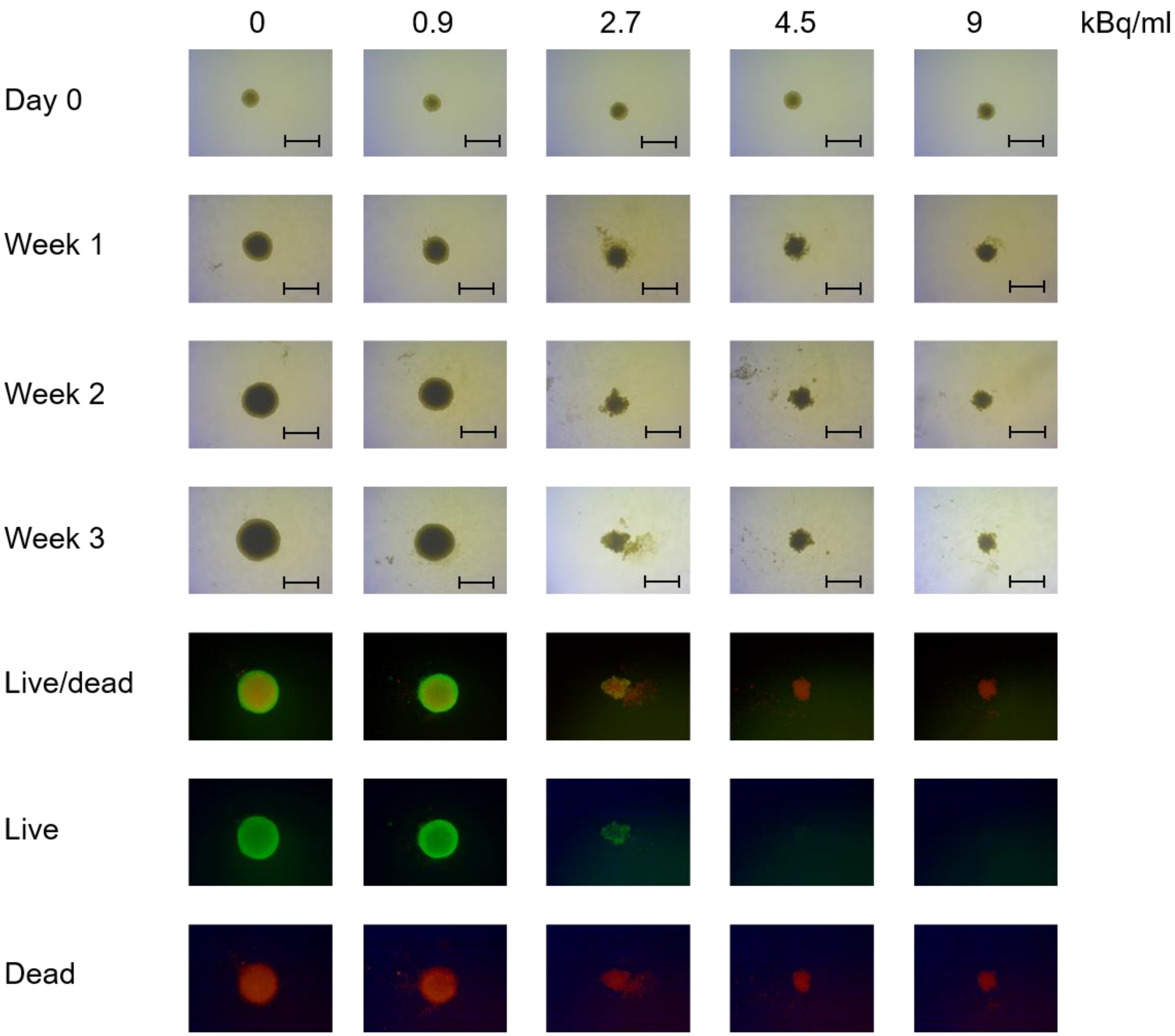

Figure S3. Representative images (4× magnification, scale bar = 500 µm) presenting the cytotoxic effect of ^{212}Pb-TCMC-TP-3 treatment for 1 h from day 0 ($8.5 \pm 0.5 \times 10^6$ µm^3) until week 3. At the experimental end point (week 3), spheroids were stained with fluorescein diacetate and propidium iodide to observe live and dead cells, respectively. All spheroid images were taken by an inverted Axiovert 200M microscope (Carl Zeiss AG) and analyzed with the AxioVision Rel. 4.8 software (Carl Zeiss AG).

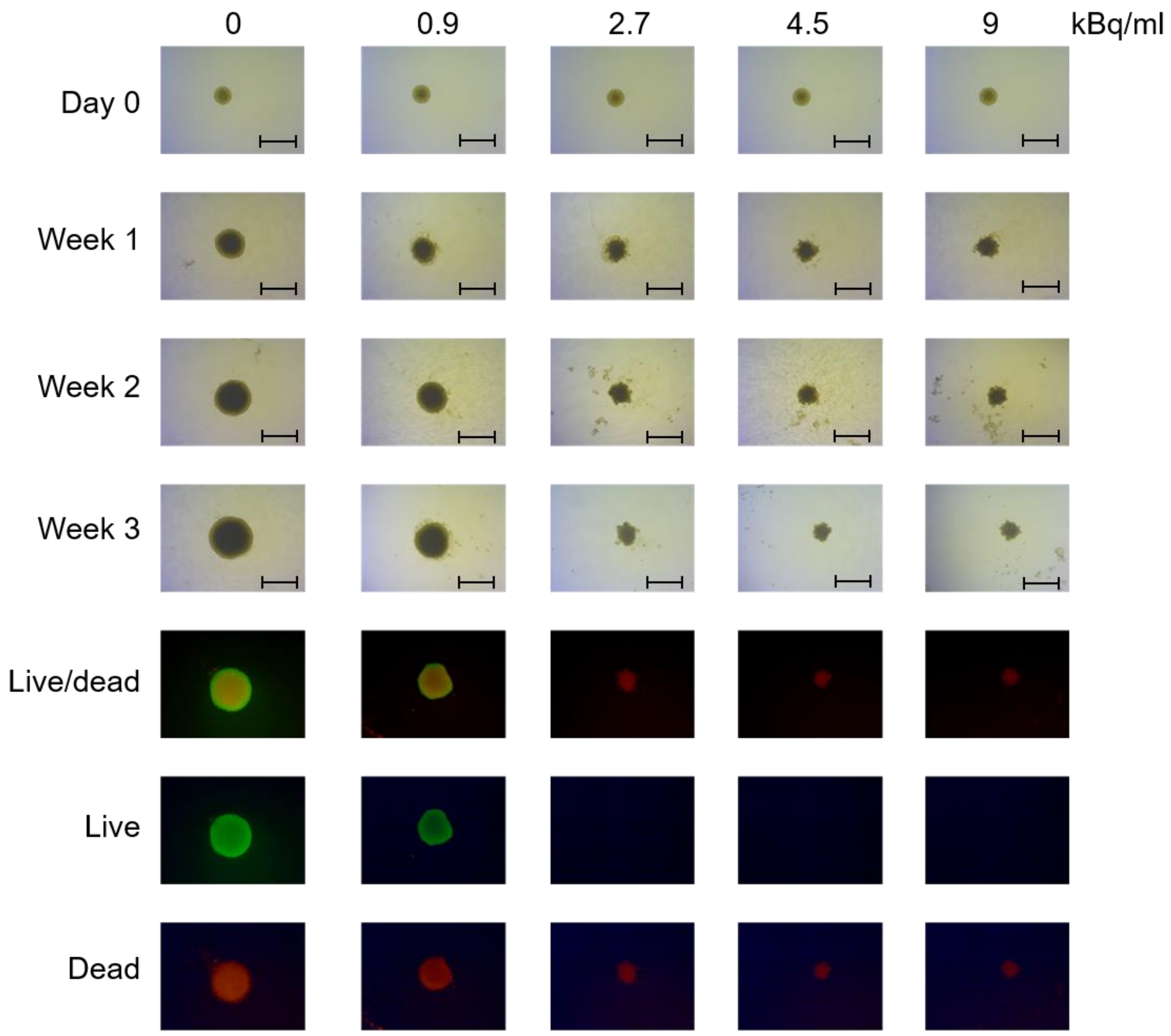

Figure S4. Representative images (4× magnification, scale bar = 500 µm) presenting the cytotoxic effect of ^{212}Pb-TCMC-TP-3 treatment for 4 h from day 0 ($8.5 \pm 0.5 \times 10^6$ µm^3) until week 3. At the experimental end point (week 3), spheroids were stained with fluorescein diacetate and propidium iodide to observe live and dead cells, respectively. All spheroid images were taken by an inverted Axiovert 200M microscope (Carl Zeiss AG) and analyzed with the AxioVision Rel. 4.8 software (Carl Zeiss AG).

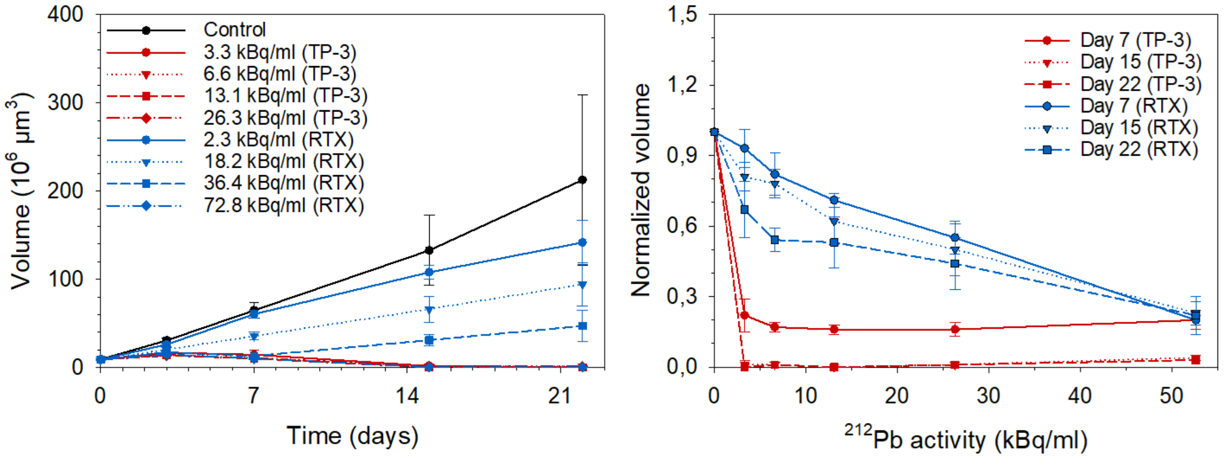

Figure S5. The influence of ^{212}Pb-TCMC-TP-3 or ^{212}Pb-TCMC-rituximab (RTX) treatment for 24 h on OHS spheroid growth after over time (left) and at increasing ^{212}Pb-activities (right).

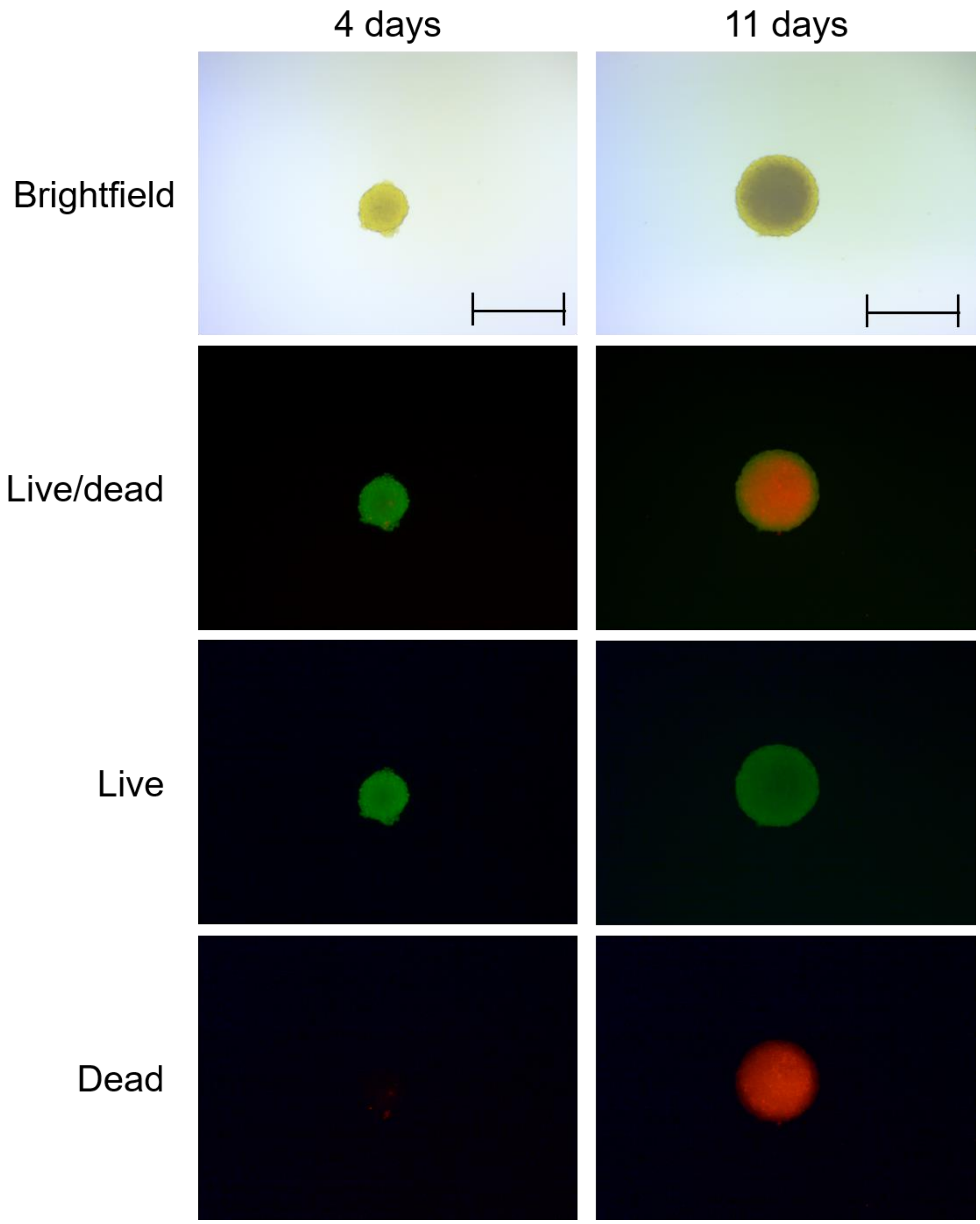

Figure S6. Representative images of untreated small (left) and large (right) spheroids 4 and 11 days after spheroids were formed from 500 OHS cells, respectively. Spheroids were stained with fluorescein diacetate and propidium iodide to observe live and dead cells, respectively, before spheroids were imaged (4x magnification) using an inverted Axiovert 200M microscope (Carl Zeiss AG, Jena, Germany) and analyzed with the AxioVision Rel. 4.8 software (Carl Zeiss AG). Scale bar = 500 µm.

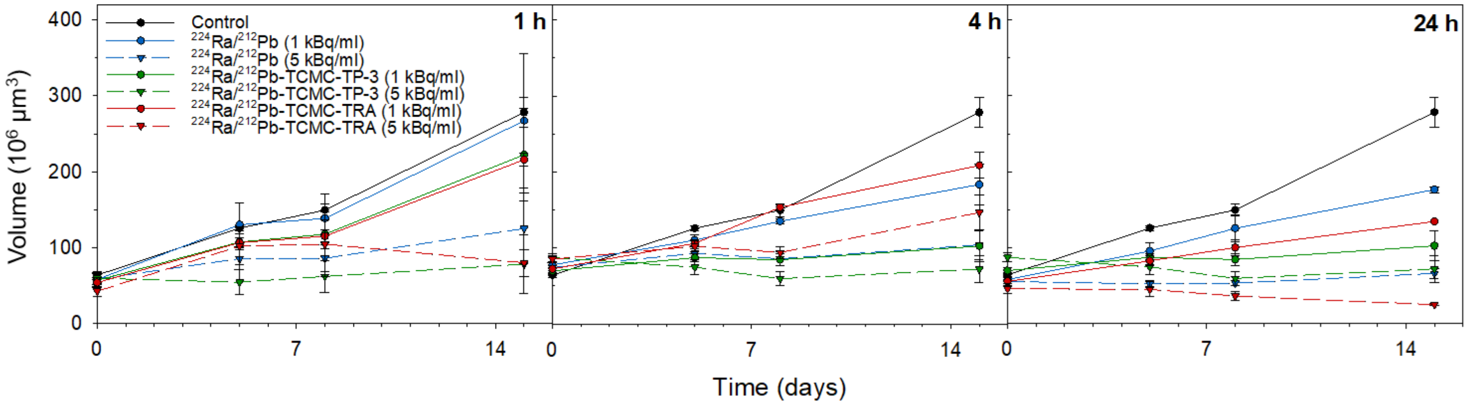

Figure S7. The influence of 1 and 5 kBq/ml $^{224}Ra/^{212}Pb$, $^{224}Ra/^{212}Pb$-TCMC-TP-3 or $^{224}Ra/^{212}Pb$-TCMC-trastuzumab (TRA) treatment for 1, 4 or 24 h on OHS spheroid growth over time. Spheroids were $60 \pm 5.0 \times 10^6$ μm^3 at day 0.
